# The Dynamics of Voluntary Force Production in Afferented Muscle Influence Involuntary Tremor

**DOI:** 10.3389/fncom.2016.00086

**Published:** 2016-08-19

**Authors:** Christopher M. Laine, Akira Nagamori, Francisco J. Valero-Cuevas

**Affiliations:** ^1^Department of Biomedical Engineering, University of Southern CaliforniaLos Angeles, CA, USA; ^2^Division of Biokinesiology and Physical Therapy, University of Southern CaliforniaLos Angeles, CA, USA

**Keywords:** tremor, force control, muscle models, closed-loop system, dynamics

## Abstract

Voluntary control of force is always marked by some degree of error and unsteadiness. Both neural and mechanical factors contribute to these fluctuations, but how they interact to produce them is poorly understood. In this study, we identify and characterize a previously undescribed neuromechanical interaction where the dynamics of voluntary force production suffice to generate involuntary tremor. Specifically, participants were asked to produce isometric force with the index finger and use visual feedback to track a sinusoidal target spanning 5–9% of each individual's maximal voluntary force level. Force fluctuations and EMG activity over the flexor digitorum superficialis (FDS) muscle were recorded and their frequency content was analyzed as a function of target phase. Force variability in either the 1–5 or 6–15 Hz frequency ranges tended to be largest at the peaks and valleys of the target sinusoid. In those same periods, FDS EMG activity was synchronized with force fluctuations. We then constructed a physiologically-realistic computer simulation in which a muscle-tendon complex was set inside of a feedback-driven control loop. Surprisingly, the model sufficed to produce phase-dependent modulation of tremor similar to that observed in humans. Further, the gain of afferent feedback from muscle spindles was critical for appropriately amplifying and shaping this tremor. We suggest that the experimentally-induced tremor may represent the response of a viscoelastic muscle-tendon system to dynamic drive, and therefore does not fall into known categories of tremor generation, such as tremorogenic descending drive, stretch-reflex loop oscillations, motor unit behavior, or mechanical resonance. Our findings motivate future efforts to understand tremor from a perspective that considers neuromechanical coupling within the context of closed-loop control. The strategy of combining experimental recordings with physiologically-sound simulations will enable thorough exploration of neural and mechanical contributions to force control in health and disease.

## Introduction

It is well known that humans cannot produce a perfectly stable force. Within the context of precise, goal-directed actions, involuntary force fluctuations can reveal clinically relevant information about neuromuscular control in disorders such as dystonia (Xia and Bush, 2007; Chu and Sanger, 2009), Parkinson's disease (Vaillancourt et al., 2001; Ko et al., 2015), bruxism (Laine et al., 2015b), and essential tremor (Héroux et al., 2010), among others. In such tasks, the nature of force variability may be influenced by both central and peripheral components of sensorimotor integration.

Unfortunately, the utility of measuring involuntary force fluctuations (i.e., tremor) within scientific or clinical settings has been limited due to the large and often ambiguous set of factors which can influence such measures. In some cases, tremor may reflect a mechanical resonance whose frequency depends on the physical characteristics of the muscle/limb in question (Lakie et al., 2012; Vernooij et al., 2013). At the same time, tremor may stem from cycles of excitation around the stretch-reflex loop (Lippold, 1970; Young and Hagbarth, 1980; Christakos et al., 2006; Erimaki and Christakos, 2008). The two mechanisms likely interact, since reflex activity is itself influenced by muscle/tendon compliance (Rack et al., 1983), limb loading (Joyce and Rack, 1974), contraction history (Gregory et al., 1998), and the temporal dynamics of force production (Xia et al., 2005).

It is clear that the specific type and extent of neuromechanical coupling influencing performance of a given task are of key importance for understanding the generation of force variability. Understanding the factors which influence dynamic force control is especially important given that this is the basis of manual dexterity during activities of daily living. However, the neural and/or mechanical origins of unintended force variability are not always clear, particularly within the context of dynamic force control.

In this study, we investigated the relationship between voluntary force production and involuntary force variability in a group of healthy adults engaged in a dynamic, isometric force tracking task. Given the various links between reflex activity, contraction dynamics, and tremor, our hypothesis was that involuntary force variability would depend upon voluntary contraction dynamics. In order to better understand the potential sources of force variability within our experimental task, we used a physiologically-realistic computer simulation to determine the sufficiency of muscle-tendon mechanics and reflex pathways to reproduce our experimental results. The simulation also allowed us to characterize the sensitivity of force variability to parameters such as reflex gain.

The significance of our study is two-fold. First, we describe a novel source of tremor along with a method for its experimental induction, and strong evidence for its origin in musculotendon dynamics. Second, the sensitivity of this tremor to both neural and mechanical factors within our simulation implies that simple force tracking tasks, such as described here, may represent a novel approach to investigating peripheral components of sensorimotor integration in health and disease.

## Methods

All procedures were approved by the institutional review board at the University of Southern California and all participants gave informed written consent prior to participation. Ten healthy participants were recruited (4 female, 6 male, aged 23–31 years) to carry out force tracking experiments.

### Physiological data

#### Task

**Figure 1:**
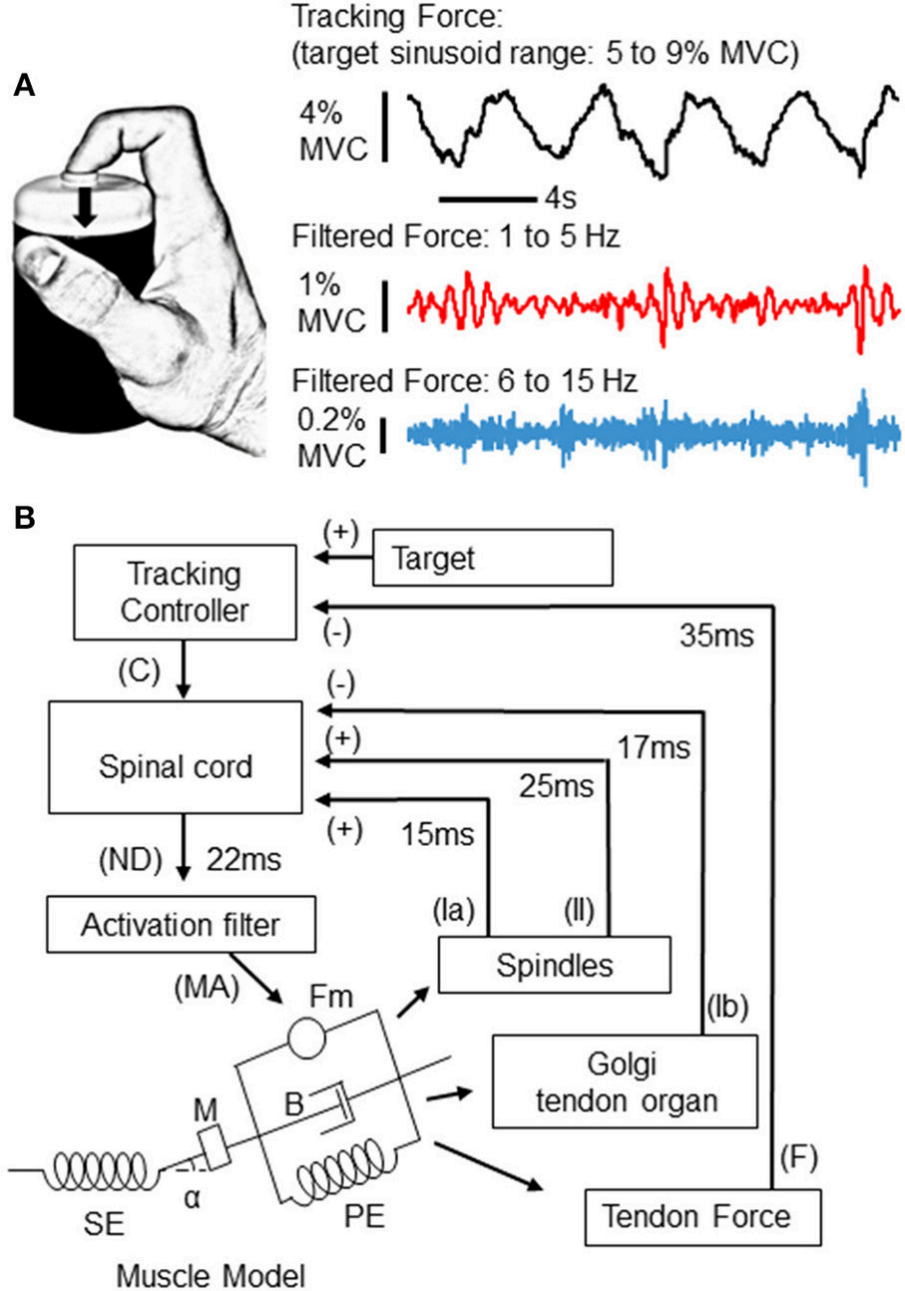
**Experimental task and simulation**. Participants were asked to produce isometric force against a small load cell with the tip of the index finger **(A)**. Using feedback of their applied force, participants tracked a 0.25 Hz sinusoid during 80 s trials. The range of the target sinusoid was 5–9% of each individual's maximum voluntary contraction (MVC) force. An example of the recorded force from one participant is depicted to the right (top trace). Below, the same force trace has been filtered into 2 different bands, a 1–5 Hz band which captures slow tremor and tracking error (middle), and a 6–15 Hz band which reveals physiological tremor and fast twitches (bottom trace). In this example, both high and low frequency force fluctuations appear to depend upon the phase of the tracked sinusoid. **(B)** Depicts the implementation of a control loop used to simulate the tracking experiment above. At the “spinal cord,” three sources of input are summated. The first two sources are proprioceptive signals from the muscle spindle (via group Ia and II afferents), and Golgi tendon organ (GTO) (via Ib afferents) models, which provide positive and negative feedback to the “spinal cord,” respectively. The third input is a tracking signal sent from a supraspinal controller. This “tracking controller” sends an output (C) which is continually updated according to the difference between the target and the force (F) produced by the muscle (see text for details). From the “spinal cord,” a neural drive (ND) signal is sent to a Hill-type muscle model via an “activation filter” which further shapes the neural drive to account for calcium dynamics within physiological muscle. The filtered muscle activation (MA) signal sent from the “activation filter” generates contraction (Fm), accounting for physical properties of the muscle-tendon complex, such as a series and parallel elastic element (SE and PE), mass (M), viscosity (B), and pennation angle (alpha). The delays and sign associated with each feedback loop are also depicted.

Participants were seated ~1 m from a 17-inch computer monitor which displayed a sinusoidal target with a vertical range representing forces from 5 to 9% of the maximum force that each individual could exert with the index finger of their self-reported dominant hand (see Figure 1A). Visual feedback of exerted force was provided in the form of a cursor which moved left to right across the computer screen for 40 s before looping back to the left. Prior to recordings, participants practiced tracking several target cycles to become familiar with the task. Each participant then tracked the 0.25 Hz sinusoidal target for two 80 s trials separated by several minutes of rest. A slow sinusoidal target is a rich behavior that is ideal for probing dynamic dependencies. For example, if tremor depended on force velocity, then tremor amplitudes would appear to follow the derivative of the target sinusoid (i.e., a cosine). If one direction of force (increasing vs. decreasing) were tied to tremor amplitudes, then tremor amplitudes would be largest along either the rising or falling phase of the target sinusoid. If the magnitude of force were most relevant, tremor amplitudes would essentially follow the target trajectory, being largest at the peaks and smallest at the valleys.

As depicted in Figure 1A, a miniature single-axis force transducer was fixed to the top of a plastic cylinder and located under the tip of the finger. Participants were asked to produce a downward force perpendicular to the force sensor, an action requiring contraction of the index finger slip of the flexor digitorum superficialis (FDS) muscle. This particular muscle and joint action were chosen because flexion at the proximal interphalangeal (PIP) joint is necessary for manipulation activities of daily living, and because this straightforward mechanical action is well suited for simulation.

### Force and EMG measurements

Surface EMG recordings were made over the distal portion of the index finger slip of the FDS muscle using an active bipolar electrode (Biometrics Ltd, Newport, UK) grounded at the wrist. Confirmation of correct electrode positioning was accomplished via palpation of the distal muscle belly (~7 cm proximal to the crease of the wrist, on the ulnar side) during index finger flexion, as well as observation of ongoing EMG signals during PIP joint flexion/extension and during our isometric task. The EMG signals were acquired at 1000 samples per second using a Biometrics DataLog system and associated software. The measurement of force, and the display of visual feedback to participants, was accomplished using custom MATLAB (The MathWorks, Natick, MA, USA) scripts to acquire force signals from a miniature load cell (ELB4-10, Measurement Specialties, Hampton, VA, USA) using a USB-DAQ (National Instruments, Austin TX, USA). The data acquisition unit sent a synchronization pulse to the biometrics system at the start of each recording. The data were analyzed offline using custom MATLAB scripts.

### Data analysis

#### Conversion of sinusoidal force to instantaneous phase

To uncover the slow, voluntary force associated with the intended target trajectory, the force produced by each participant was low-pass filtered at 0.5 Hz. Using the Hilbert transform, the instantaneous phase of this tracking force was calculated and expressed in degrees (0–360°) over the course of each target cycle. This conversion was useful since instantaneous phase holds information about the actual dynamics of force production at a given time, regardless of tracking error. Although tracking error was not a focus of this investigation, it was still important to eliminate poorly tracked target cycles. Target cycles in which the absolute tracking error exceeded 4% of a participant's MVC level at any time point were excluded from all further analysis.

#### Calculation of instantaneous tremor amplitude

To quantify the presence of involuntary force fluctuations, the sinusoidal force trajectories produced by each participant were filtered into two different frequency bands.

First, we investigated the presence and magnitude of force fluctuations at high frequencies (>6 Hz), which cover the frequency range of physiological tremor (Lippold, 1970; Elble and Randall, 1976; Burne et al., 1984; Christakos et al., 2006). These force fluctuations were extracted by band-pass-filtering the force produced by each individual between 6 and 15 Hz (zero-phase, 4th order Butterworth filter). Beyond about 15 Hz, the amplitude of force fluctuations is essentially negligible due to the low-pass filtering effects of tissue (finger pad, tendon) and muscle.

Second, we quantified slow (1–5 Hz) force fluctuations. Generally, slow fluctuations in force stem from changes in the overall drive to motor neurons (Allum et al., 1978; De Luca et al., 1982; Miall et al., 1993; Slifkin et al., 2000; Squeri et al., 2010). These slow fluctuations do include voluntary correction of tracking errors, but our analysis focused on fluctuations that were consistently present at particular phases of the target cycle, and therefore reflect an involuntary process. To extract these fluctuations, we used a 1–5 Hz band-pass-filter (zero phase, 4th order Butterworth filter).

An example of band-pass filtered force traces in relation to the target sinusoid is depicted in Figure 1A (right).

#### Calculation of tremor modulation as a function of tracking phase

As described above, the tracking force produced over time by each participant was converted to a trace of instantaneous phase angles where each complete target cycle was represented as a progression from 0 to 360°. Each cycle was then divided into 36 phase-bins (each representing 10°). Again, it should be noted that all analyses are based on the temporal dynamics of the force produced by the participants and not on the displayed target. This renders any positive or negative tracking lags irrelevant [although they would be minimal given the highly feed-forward nature of this type of task (Erimaki et al., 2013)]. To examine the relationship between tracking phase and force variability, we first converted each band-pass filtered force signal into an instantaneous amplitude signal by rectification and smoothing with a 200 ms Gaussian window. The magnitude of the resulting smoothed/rectified signal also serves as a simple estimation of instantaneous variance within the specified frequency band, given the equivalence between total signal power (in frequency domain) and total signal variance (in time domain) (i.e., Parseval's theorem). We then found the sum of the filtered/rectified force values associated with each phase interval, and divided each by the integral of the filtered/rectified force trace. This procedure gives the relative proportion of total force variability (within the specified frequency band) associated with each 10° phase interval of the target cycle.

Under the null hypothesis, the proportion of force variability in each phase bin does not depend on the phase progression of the target cycle. Thus each 10° phase bin would be expected to show about 2.8% (100%/36 bins) of the total force variance. To test the null hypothesis, we compared our recorded proportions to a phase-randomized distribution generated directly from the recorded data (i.e., shuffled versions of our own data). We constructed these shuffled distributions of proportion values by randomly selecting a different 10° phase bin in each tracked target cycle, and then calculating the proportion of total force variability, as previously described. The process was repeated 5000 times, creating a distribution of shuffled proportion values which allowed us to determine a 95% confidence interval. Proportions falling outside of this interval would then represent statistically significant deviations from chance level. Our use of a Monte–Carlo method provides a direct, conservative, and assumption-free statistical analysis. Similar methods are often used in neuroscience, where analysis of real vs. shuffled/randomized neural activity is common (Perkel et al., 1967; Tam et al., 1988; Türker et al., 1996; Rivlin-Etzion et al., 2006; Laine et al., 2012). In our case, alternative methods such as testing for differences between individual phase bins, would be ill-suited for identifying the timing of tremor modulation with respect to the target phase, and would also not account for the fact that the proportion within each phase bin is not strictly an independent measurement.

The above methodology was applied to individual participants. To evaluate the population as a whole, the proportions for each phase bin were averaged across individuals. As a statistical evaluation, we calculated, for each phase bin, the number of individuals whose tremor proportion fell above or below the 95% confidence interval. For any given phase bin, a 5% error rate might be expected. Since our analysis included 10 individuals, it could be expected that at least one may have exceeded the confidence level purely by chance. However, the binomial probability that 2 of 10 individuals should show a (false positive) significance at the 95% confidence level is 0.015. For this reason, our population significance level was set to 0.015, or 2 out of 10 individuals, for our consistency analysis.

In addition to analyzing the proportion of force variance in each phase interval, we also calculated the cross-cycle average tremor amplitude in each phase interval. This analysis yielded an amplitude profile for each individual (and frequency band), similar to the proportion profiles described above. We then recorded the maximum and minimum values observed in the amplitude profile of each individual, regardless of the particular phase at which these values were found. This allowed us to evaluate the actual extent of tremor amplitude modulation, uncoupled from any particular pattern of tremor modulation across target phases.

#### Force to EMG coherence across target phases

Coherence is a frequency-domain measure of synchronization (primarily phase-locking) between signals, and is bounded between 0 (no correlation between signals) and 1 (perfect linear correlation). Coherence between rectified EMG activity and force is useful for identifying the frequency content of force-relevant neural drive to muscles, since action potential shapes/sizes and other recording artifacts only influence the EMG spectrum, but would not be synchronized with force. In addition, coherence between FDS activity and force tremor provides validation that our simulation of a dynamically activated FDS muscle is appropriate for exploring the potential origins of recorded force fluctuations.

To calculate EMG to force coherence, the force and EMG signals were concatenated across all trials from all subjects to form two long signals. These signals were then converted to time-frequency-representations (TFRs) via wavelet analysis. We chose a wavelet approach so that we could precisely determine which frequencies of force were synchronized with EMG, and at what times. The technique is common where temporal variation of spectral power or synchronization is of interest (e.g., Siemionow et al., 2010; Tscharner et al., 2011). This was accomplished through convolution of each original signal *x(t)* with a Gaussian-windowed complex sinusoid (a Morlet wavelet), the duration of which was set to span 3 cycles of each frequency (f) from 1 to 20 Hz. The process can be expressed by the following formula:

$$TFR\left(t,f\right)=\int x\left(t\right)\frac{1}{\sigma \sqrt{2\pi }}e^{-}\frac{{\left(t-\tau \right)}^{2}}{2\sigma^{2}}e^{-j2\pi f(t-\tau )}dt$$

where the standard deviation (σ) of the Gaussian window is set to 3/(2πf). The force trace (band-pass filtered between 1 and 20 Hz) as well as the EMG activity (rectified, normalized per subject to have unit variance) were thus converted to complex-valued TFRs (herein defined as TFR_Force and TFR_EMG, respectively). The spectral power of each signal can be calculated as follows:

$$\begin{array}{lll} \text{Power}\_\text{Force}\left(\text{t},\text{ f}\right) & = & \text{TFR}\_\text{Force}\left(\text{t},\text{ f}\right)•\text{conj}\left(\text{TFR}\_\text{Force}\left(\text{t},\text{ f}\right)\right)\\ \text{Power}\_\text{EMG}\left(\text{t},\text{ f}\right) & = & \text{TFR}\_\text{EMG}\left(\text{t},\text{ f}\right)•\text{conj}\left(\text{TFR}\_\text{EMG}\left(\text{t},\text{ f}\right)\right) \end{array}$$

Where conj refers to the complex conjugate.

Likewise, the time-frequency cross-spectrum can then be defined as:

$$\begin{array}{l} \text{TFR}\_\text{cspec}\left(\text{t},\text{ f}\right)=\text{TFR}\_\text{Force}\left(\text{t},\text{ f}\right)•\text{conj}\left(\text{TFR}\_\text{EMG}\left(\text{t},\text{ f}\right)\right) \end{array}$$

The time course of coherence can then be calculated per frequency as:

$$\begin{array}{l} \text{TFR}\_\text{Coherence}\left(\text{f}\right)=\\ \;\frac{|\text{ TFR}\_\text{cspec }(\text{f})\;*\text{ W }{|}^{\wedge }2}{\left(\text{Power}\_\text{Force}\left(\text{f}\right)*\text{W}\right)\;•\;\left(\text{Power}\_\text{EMG}\;(\text{f})*\text{ W}\right)} \end{array}$$

Where the term ^*^W represents convolution of the indicated time series with a rectangular window (W), the duration of which was set per frequency to be 7/f. The multiplication and division in the above equation are simply element-by-element operations on the time series data.

Prior to further analysis, coherence values were normalized using Fisher's r-to-z transform Fz = atanh(√C) where C is the coherence at a given time-frequency point (Benignus, 1969).

Because of the short time scales involved in the calculation of wavelet coherence, it is best to recast coherence values as a statistical deviation from chance level. Here, the chance level was derived empirically by recalculating the time-frequency coherence after reversing the concatenated EMG signal in time. This causes EMG signals from one participant to be tested for coherence with force traces produced by a different participant, and completely misaligns the signals with respect to the phase progression of the sinusoidal target. The actual coherence values for each frequency were then converted to standard *Z*-scores with respect to the distribution of coherence values obtained from the “fake” time series. This method helps to emphasize any synchronization which varies significantly across target phases. Values > 1.65 (the one-sided 95% confidence level for a *Z*-test) indicate that the time-localized coherence between EMG and force was greater than expected by chance at a given phase and frequency.

### Simulations

#### Closed-loop control overview

We used a computational model of an afferented muscle to study the dependence of tremor on the dynamics of force production. A schematic diagram of the feedback-driven control loop is shown in Figure 1B. Briefly, a Hill-type muscle-tendon model was driven by a neural activation signal to produce force under isometric conditions. The simulation was intended to approximate the action of the FDS muscle in our experimental data. The muscle-tendon model describes changes in force as well as the magnitude and rate of associated changes in the length of the muscle fascicle and tendon, accounting for their viscoelastic properties. Our simulation includes two spinal proprioceptive systems; the muscle spindle and the GTO. Upon muscle fiber lengthening, the muscle spindle sends excitatory feedback through primary (Ia) and secondary (II) afferent fibers proportional to eccentric changes in muscle fiber length and velocity; while GTOs send inhibitory feedback (Ib) proportional to the force in the tendon. A tracking controller, whose operation includes conduction and synaptic delays appropriate for a transcortical loop (Lourenço et al., 2006; Pruszynski et al., 2011; Sohn et al., 2015), sends a command signal (C) to the “spinal cord” which is corrected at each time step according to the difference between the target force level and the actual force output from the muscle. This tracking control signal simply ensured that the afferented muscle-tendon model could follow the target force trajectory, and is not intended to model a specific neural pathway, or to recreate human visuomotor or voluntary tracking behaviors. Signals from the tracking controller, muscle spindle, and GTO, are integrated at the “spinal cord” to generate the α-motoneuron drive to the lumped-parameter muscle model. This neural drive (ND) at each ms (t) can be expressed in the following form:

$$\begin{array}{l} \text{ND}\left(\text{t}\right)\;=\text{ Ia}\left(\text{t}-15\right)+\text{II}\left(\text{t}-25\right)-\text{Ib}\left(\text{t}-17\right)+\text{C}\left(\text{t}\right) \end{array}$$

The output of the tracking controller (C) is calculated as:

$$\begin{array}{l} \text{C}\left(\text{t}\right)\;=\text{ C }\left(\text{t}-1\right)\;+\text{k}•\left(\text{Target}\left(\text{t}\right)-\text{F}\left(\text{t}-35\right)\right) \end{array}$$

where F is the force on the tendon and k is a constant. Note that the above represents a simple “iterative learning control” (ILC) scheme (Wang et al., 2009).

To translate the neural drive into force, the signal was delayed by an additional 22 ms before reaching the muscle fibers to account for conduction time along efferent fibers. At the muscle, the signal was passed through an “activation filter” which shapes the signal to account for calcium dynamics in physiological muscle. Finally, the muscle-tendon model converts this muscle activation signal to the force output of the tendon. In this simulation, the delays for each pathway have been matched to physiological recordings from humans and reflex latencies from the FDS muscle in particular (Lourenço et al., 2006).

The muscle model, muscle spindle, and GTO elements of this control loop have been published previously by various groups and will be described and referenced individually below.

### Control loop elements

#### Muscle model

Our Hill-type muscle-tendon model and its mathematical derivation were adopted from previous literature (He et al., 1991; Brown et al., 1996). The schematic diagram of this muscle-tendon model is presented in Figure 1B. The muscle fascicle consists of a mass (M), two passive elastic elements (PE in Figure 1B), a viscous element (B), and a contractile element (Fm), which is connected with a pennation angle (α) to a series elastic element (SE) representing tendon and aponeurousis.

The contractile element generates muscle force as a fraction of the maximal force that the muscle is capable of producing. This is defined as the product of its physiological cross-sectional area and a constant factor (45 N/cm^2^) (Holzbaur et al., 2005). Two parallel elastic elements characterize passive behaviors of muscle fascicles. The first (non-linear) spring acts against stretch of muscle fascicle, while the second (linear) spring resists compression (Brown et al., 1996). The series elastic element (SE) shown in Figure 1B is a lumped non-linear spring model of tendon and aponeurosis. The force produced by this element in relation to the length of the tendon has been implemented as in Brown et al. (1996). The contraction dynamics within the muscle-tendon unit are modeled as a second-order differential equation (He et al., 1991).

Taking the above factors into account, the output force function (F) can be summarized as follows

$$\begin{array}{l} \text{F}\left(\text{t}\right)=\text{MA}\left(\text{t}\right)•\text{FL}\left(\text{t}\right)•\text{FV}\left(\text{t}\right)+\text{F}\_\text{PE}1\left(\text{t}\right)+\text{F}\_\text{PE}2\left(\text{t}\right)\\ +\;\left(\text{B}•\text{v}\left(\text{t}\right)\right)+\left(\text{a}\left(\text{t}\right)•\text{M}\right) \end{array}$$

Where MA is the muscle activation (the output of the activation filter), FL is the force-length function, FV is the force-velocity function, F_PE1 and F_PE2 are the forces produced by the two elastic passive elements in the model, v(t) and a(t) are the velocity and acceleration of muscle fiber contraction, and the muscle mass (M) and viscosity (B) are constants.

Because the force produced by human participants ranged from 5 to 9% of maximal effort, we applied the same forces to the simulated FDS muscle. Given our focus on understanding the general nature of tremor modulation by dynamic force production, it was not necessary to calculate the precise, isolated contribution of the FDS muscle to the generation of index finger force in our experimental task. To simulate the FDS muscle, architectural parameters were set (Table 1) according to published anatomical data (Lieber et al., 1991; Holzbaur et al., 2005). For our purposes, the muscle fibers of the FDS muscle associated with the tendon acting on PIP joint of the index finger were combined into a single belly for simplicity.

**Table 1:** **Architectural parameters of the slip to the index finger of the flexor digitorum superficialis (FDS) muscle**.

Mass (g)	12
Optimal fascicle length (cm)	8.4
Resting fascicle length (cm)	6.8
Tendon slack length (cm)	27.5
Pennation angle (°)	6
Cross-sectional area per head (cm^2^)	1.7

#### Muscle spindle model

The muscle spindle model employed in this study is adapted from Mileusnic and Loeb (2006). This computational model was chosen because it is both physiologically realistic and, at the same time, is immediately compatible with the inputs and outputs of the other elements within our control loop. The model comprises three types of intrafusal fibers, namely, the bag_1_, bag_2_, and chain fibers, all of which are modeled as a second-order mechanical system (a mass, a viscous element, and parallel and series elastic elements), similar to a Hill-type muscle-tendon model. Each of the intrafusal fibers receives input describing the muscle fascicle length, velocity, and acceleration, as well as a fiber-type-specific fusimotor activation signal (dynamic or static). In this study, fusimotor activation was set to be constant during each simulation run. The fusimotor gains tested were 75, 150, and 350. Functionally, these are arbitrary units, but can be expressed conceptually as pulses per second. We chose to define our baseline value as 75, since this is near the previously published value of 70 (Mileusnic and Loeb, 2006), and we varied that parameter because fusimotor drive is known to depend upon task and individual psychology (Ribot et al., 1986; Ribot-Ciscar et al., 2000, 2009; Hospod et al., 2007). Because fusimotor drive is modified by the nature of the task independently of (and even without) α-motoneuron firing (neural drive, in our model), we chose not to assume obligatory α-γ coactivation. It is true that mechanisms other than fusimotor drive may change the effective gain of afferent activity (e.g., presynaptic inhibition). Here, variation in fusimotor drive is not only a likely physiological occurrence, but also serves to more generally represent the overall gain of spindle feedback to motor neurons. For integration with the feedback control loop, the final outputs of the spindle model were normalized to fall between 0 and 1.

#### Golgi tendon organ model

The GTO model was adopted from Elias et al. (2014). This GTO model presents the overall behavior of a population of Ib fibers. It was placed in series with tendon, so that it receives tendon force as an input. The force was then converted into Ib fiber output. The transfer function described in Elias et al. (2014) was implemented using the c2d function in MATLAB. The Ib fiber output was scaled between 0 and 1, as was carried out for the spindle outputs.

#### Activation filter

The activation filter adjusts the neural drive signal to account for the effects of calcium dynamics (release and reuptake) on cross-bridge formation, as described in Song et al. (2008). The resulting muscle activation signal (MA in Figure 1B) is the “effective” drive delivered to the muscle model.

### Simulation analysis

Force tracking was simulated for 128 s with new values for each output parameter derived every ms. To be certain that only consistent, steady-state behavior was analyzed, only the last 30 cycles were used for analysis. The muscle forces produced by the simulation were analyzed in the same way as the experimentally recorded force tremor, providing a direct comparison.

## Results

### Tremor during force tracking

**Figure 2:**
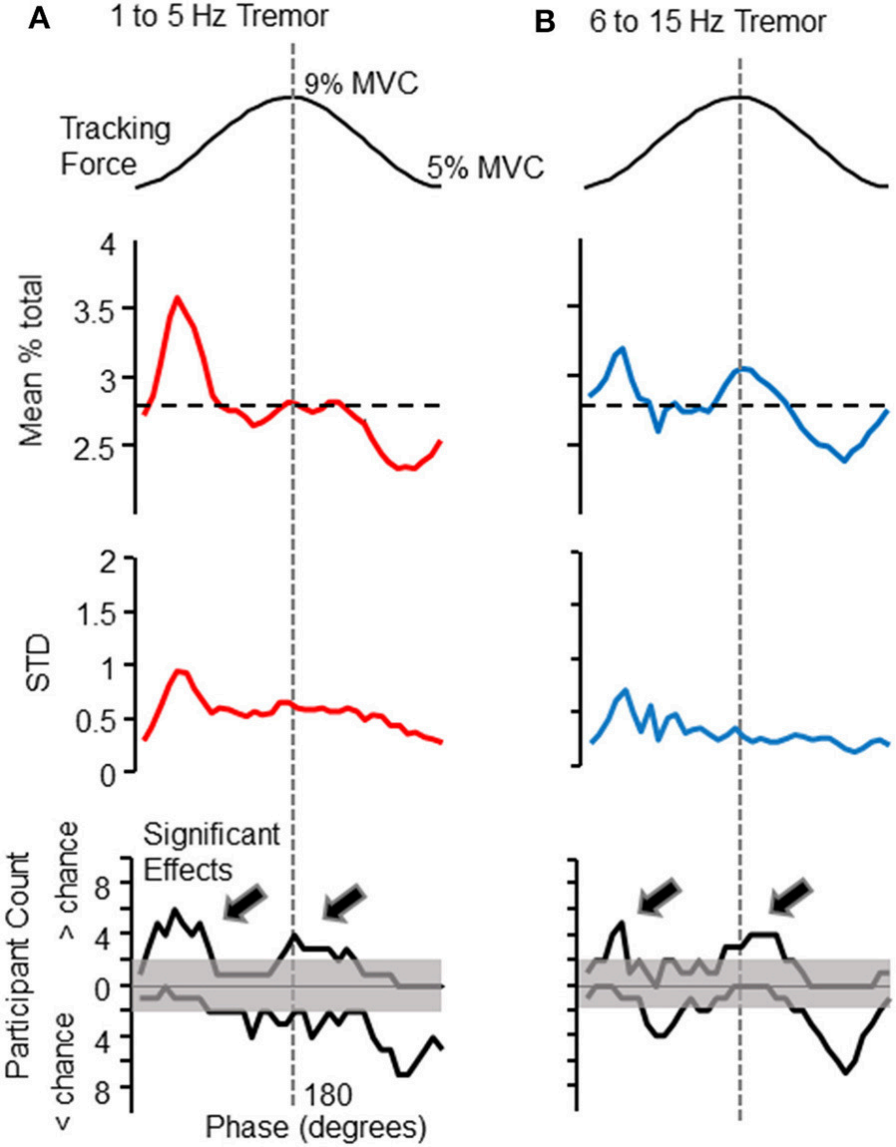
**Phase-dependent modulation of tremor amplitude**. The amplitude of force variability within the 1–5 and 6–15 Hz frequency bands depended upon the phase of the target cycle (shown at the top of each column, for reference). The first data panel of **(A)** (left column) shows the mean proportion of total force variability (within the 1–5 Hz band) observed in each 10° phase bin, calculated over all participants. The same panel of the right column **(B)** shows this analysis for 6–15 Hz force fluctuations. The horizontal dashed line in each figure represents the proportion expected per bin if tremor amplitude were constant across target phases. The 1–5 Hz frequency band shows a clear peak at the initiation of the rising phase of the target sinusoid. A similar profile was observed for 6–15 Hz force fluctuations, but with a more pronounced tremor amplification at the peak of the target sinusoid. Below these grand averages are depicted the cross-participant standard deviation for each phase bin. The cross-participant variability is generally highest at the valley of the target sinusoid, when bursts of high amplitude tremor were more likely to occur (as can be seen in Figure 1A). The bottom panels are histograms which depict the number of individuals (out of 10), whose tremor profiles deviated significantly from chance level at each phase-bin, as determined by a Monte-Carlo test (see text for details). In these histograms, counts above the 0 line record the number of individuals who displayed greater than chance-level proportions. Counts below 0 indicate the number of individuals showing lower tremor than expected by chance at each phase. Note that these “less than chance” histogram counts are an effect of other phases showing high proportions of tremor, and do not indicate suppression of ongoing tremor, which was never observed. Histogram counts exceeding the shaded region indicate that significant effects at a given phase were more consistent across individuals than could have occurred by chance (according to a binomial test). The histograms show that the modulation of tremor by target phase was fairly consistent across individuals. The arrows on each histogram (**A,B** bottom), emphasize that tremor was most often larger than expected at the peaks and valleys of the target sinusoid, for both frequency bands.

The phase of voluntary force modulation influenced both low (1–5 Hz) and high (6–15 Hz) frequency bands of involuntary tremor. For reference, the top panels of Figures 2A,B show the target isometric force sinusoid, which spanned from 5 to 9% of each individual's maximum voluntary force level. The panels immediately below the target in Figures 2A,B show the proportion of total tremor variance associated with each phase of the target sinusoid. The proportion of total force variance accumulated in each 10° phase bin is shown for low (Figure 2A, upper trace) and high (Figure 2B, upper trace) frequency bands. Each plot represents a grand average over all 10 participants, who together tracked a total of 357 target cycles. The largest tremor in either frequency band was observed at the beginning of the rising phase of the target. A second time period of increased tremor amplitude appeared at or slightly after the peak of the target cycle, mainly for high frequency tremor. The dashed line indicates the proportion of variance to be expected in each phase bin if force variability were evenly distributed over all phase bins.

Below these average tremor profiles are the cross-participant standard deviations associated with the mean proportions in the traces above. Variance across participants was highest at the base of the target sinusoid, where bursts of tremor (e.g., Figure 1) were often observed. The bottom panels of Figures 2A,B depict the number of participants (out of 10 total) whose tremor profiles showed statistically larger (above 0 line) or smaller (below 0 line) proportions in each phase bin than expected by chance (as determined by a Monte–Carlo test, as previously described). The shaded region marks the number of participants that may have been expected to show significant effects by chance. That is, histogram counts exceeding the upper limits of the shaded range represent a consistent amplification of tremor occurred across the population of participants. Histogram counts below the 0 line are caused by the high proportions observed in other phase bins and do not represent suppression of tremor, which was never observed. Again, column A shows results for 1–5 Hz force fluctuations and column B shows results for 6–15 Hz force fluctuations. For both frequency bands, a significant population effect was observed at the beginning of the rising phase of the target sinusoid, and at the beginning of the falling phase.

### EMG to force coherence

**Figure 3:**
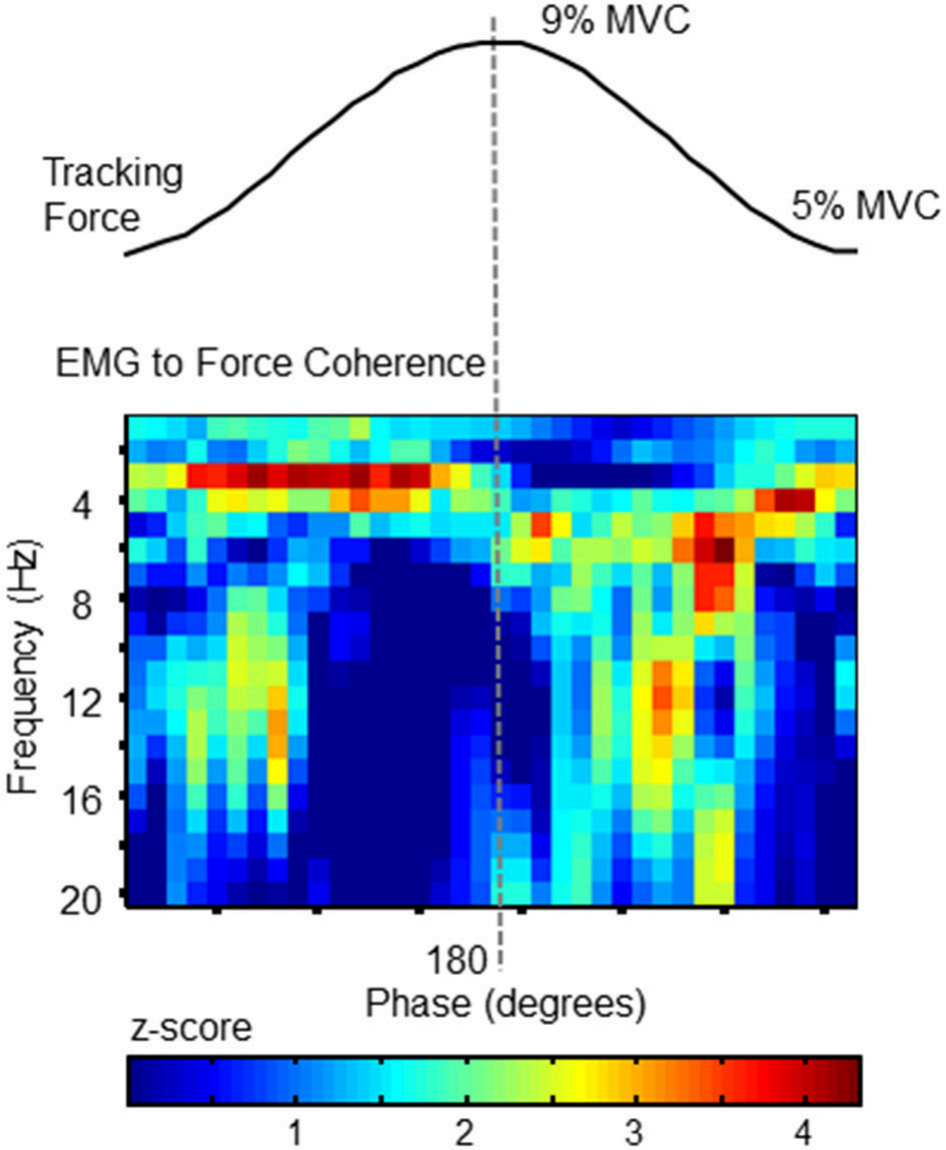
**EMG to Force Coherence**. To confirm a correlation between force fluctuations and muscle activity, coherence between FDS EMG activity and force was analyzed as a function of target-phase (x axis) and frequency (y-axis). The statistical magnitude of coherence (z-score with respect chance-level) is represented by the color of each pixel. Values >1.65 indicate statistically greater coupling than expected by chance. Each phase-frequency pixel represents the average coherence observed at that specific pixel, calculated over 357 tracked target cycles. The pattern of coupling is similar to the behavior of force fluctuations (as shown in Figure 2). This plot confirms that FDS activity corresponds with phase-dependent force fluctuations.

To confirm that the cross-cycle modulation of force variability was also reflected in the activation of the FDS muscle, we calculated EMG-to-force coherence. Using wavelet coherence, we were able to examine the coupling between signals at each frequency, and at each phase of the target cycle. The statistical magnitude of coherence (*z*-score with respect chance-level) shown in Figure 3 for each time-frequency pixel was calculated from the full data set (all 357 tracked cycles). Pixels with values greater than 1.65 can be considered as significant at the 95% confidence level.

Importantly, the coupling between EMG and force signals closely resembles the phase progression of force tremor amplitudes, and reflects the same frequency profile. The phase-related modulation of coherence demonstrates temporal variation in synchronization between signals, which would be expected if the frequency content of neural drive depended on the phase progression of the tracking action.

### Actual tremor amplitudes during force tracking

**Figure 4:**
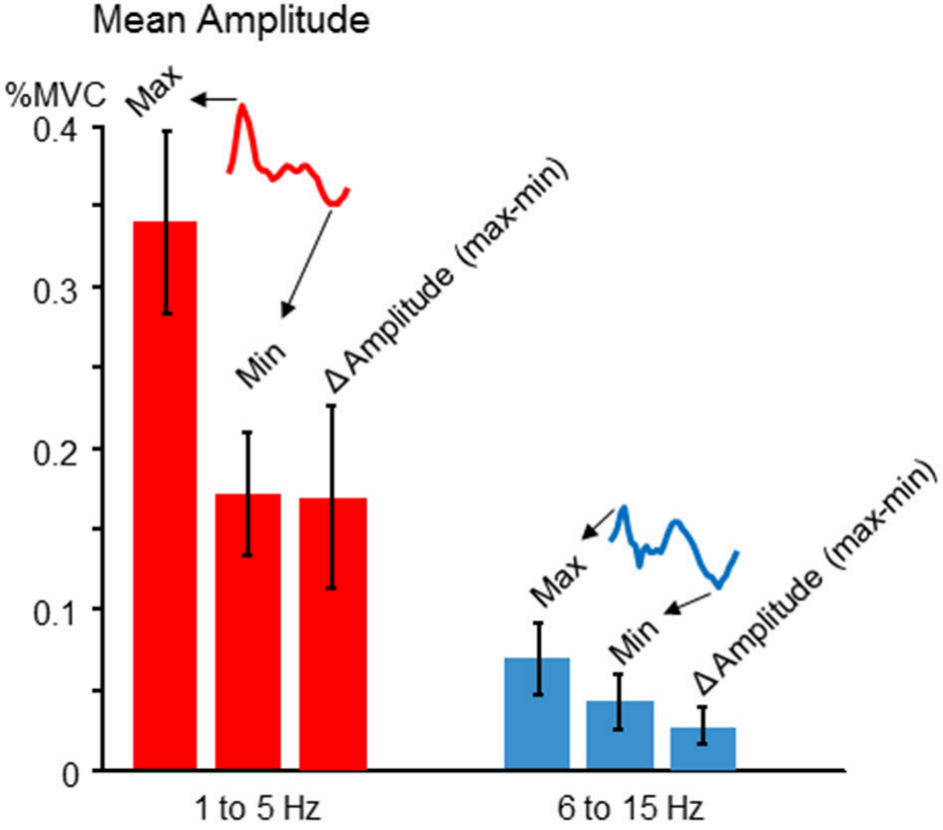
**Tremor modulation amplitude**. For each participant, the average cross-cycle tremor amplitude was calculated for each phase of the target sinusoid. The resulting amplitude profile was characterized (for both 1–5 and 6–15 Hz frequency bands) in terms of the maximum tremor amplitude, the minimum amplitude, and the difference between the two, which indexes the average amplitude modulation across one target cycle. The cross-participant mean amplitudes for each feature (regardless of the precise phase at which they occurred for each individual) are depicted by the red (low frequency) and blue (high frequency) bars. The error bars show the cross-participant standard deviations for these features. For both frequencies, there was a nearly two-fold modulation of tremor amplitude across a target cycle, on average.

Although our study is primarily focused on the modulation pattern of normalized tremor amplitudes as a function of voluntary force dynamics, it is also important to address actual tremor amplitudes, and the extent of amplitude modulation across target phases. Since the pattern of tremor modulation could vary across individuals (described below), we chose to record the maximum, minimum, and Δ amplitude (max-min). The latter was important for better comparability with our simulation results, since our simulation does not contain noise or ongoing physiological tremor, both of which are typically present in human participants. In general, we found tremor amplitudes fluctuated by a factor of about 2 over the course of a target cycle. Figure 4 shows the mean and cross-participant SD for each measure.

### Tremor modulation across different individuals

**Figure 5:**
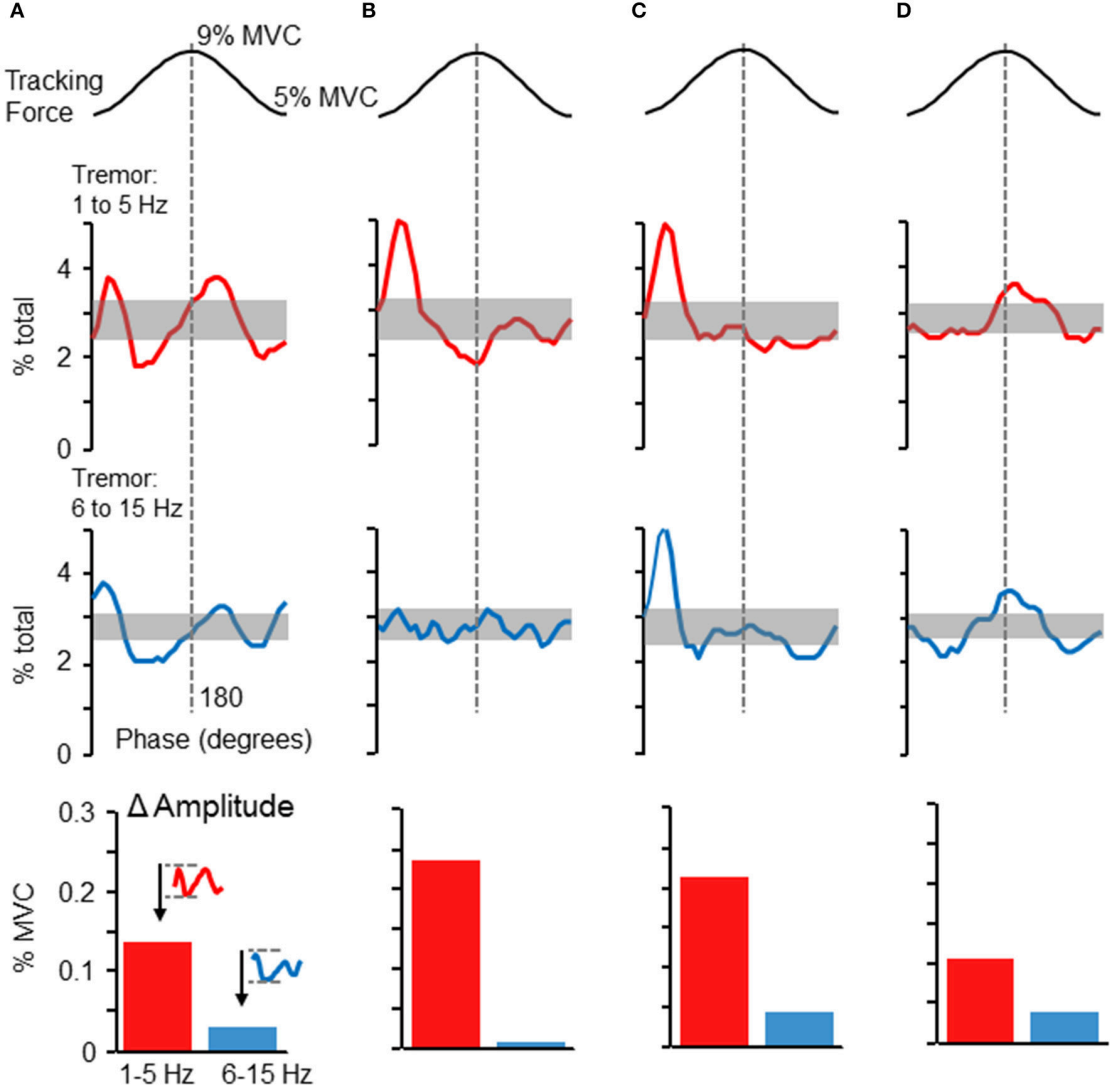
**Phase-dependent tremor modulation varies across individuals**. Tremor profiles were often variable across individual participants. Panels **(A–D)** depict the dependence of high frequency (bottom traces) and low frequency (top traces) force variability on target phase. Panels **(A–D)** represent profiles from four individuals. The shaded regions show the 95% confidence interval, as derived by a Monte–Carlo test. Tremor in either frequency range tended to be largest at the initiation of the rising phase of the target sinusoid and/or at the peak of the target sinusoid. At the bottom of each column, the average cross-cycle amplitude modulation (maximum-minimum amplitude) is plotted for the 1–5 and 6–15 Hz frequency bands.

Although the phase-dependent modulation of force variability shown in Figure 2 was representative of the population overall, tremor profiles did vary across individuals. Figures 5A–D depicts the tremor proportion profiles for high and low frequency bands in 4 individuals whose profiles differed from each other. Overall, most participants showed some degree of increased tremor (in either frequency band) at the peaks and/or valleys of the target sinusoid. The modulation of tremor amplitudes in these individuals, shown in the bar graphs at the bottom of each column, indicate that 1–5 Hz tremor amplitude was consistently higher than 6–15 Hz tremor amplitude, but the ratio could vary across individuals.

### Simulation results

**Figure 6:**
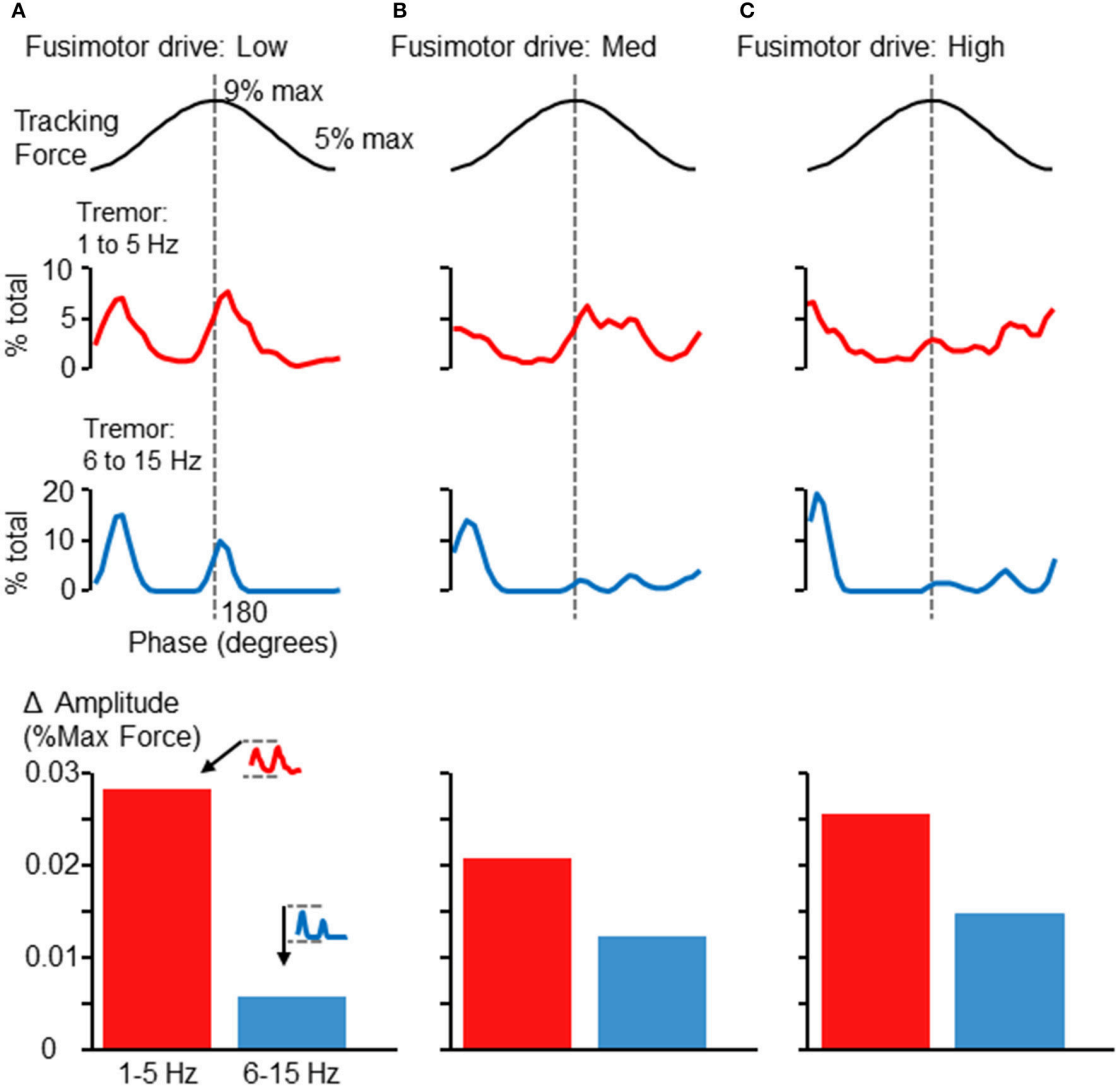
**Phase-dependent force tremor in simulation**. The simulated FDS muscle activity produced target-phase dependent tremor, as observed in human participants (Figures 2, 5). Panels **(A–C)** show the proportion of within-band force variability observed at each 10° phase bin when the simulation was run using different levels of fusimotor drive: low **(A)**, medium **(B)**, and high **(C)**. As before, the top traces show 1–5 Hz force variability and the lower traces show 6–15 Hz force variability. Alteration of fusimotor drive in this simulation was able to alter the phase-dependent modulation of tremor in both frequency bands. At the bottom of each column, the average extent of cross-cycle amplitude modulation is depicted, as in Figure 5. While changes in fusimotor drive did somewhat alter the extent of low-frequency tremor amplitude modulation, the effects were greatest on the 6–15 Hz tremor, which roughly tripled as fusimotor drive was increased from low to high. Also, the relationship between high and low frequency amplitude modulation is similar to that observed in the experimental data (Figures 4, 5, bottom).

By embedding a modeled FDS muscle within a feedback-driven control loop, we were able to simulate the force tracking experiment carried out by human participants. Surprisingly, this simple model of an afferented FDS muscle sufficed to produce much the same pattern of tremor modulation in relation to the 0.25 Hz sinusoid as seen in Figures 2, 5. Figures 6A–C shows the modulation of tremor obtained when the simulation was run using low, medium, and high fusimotor drive. Adjusting the fusimotor drive, and thus, the gain of afferent feedback from the muscle spindle, could produce variation in simulation results similar to the type of variation observed across different subjects (e.g., compare Figure 5A with Figure 6A, or Figure 5C with Figure 6C). Force fluctuations near the valley of the sinusoidal target were present in all cases, although the fluctuations occurring at the peak of the sinusoid was reduced as the afferent gain was increased. As with the experimental data, the rising and falling phases of the target sinusoid did not appear to be associated with consistent changes in tremor activity. At the bottom of each column in Figure 6 are bar graphs showing the average extent of amplitude modulation. Since the minimum amplitude was nearly 0 in all cases, these bars also represent the average maximum amplitude across phases as well. Of particular importance is the fact that increasing fusimotor gain resulted in a doubling (B) and tripling (C) of high frequency tremor amplitudes, as compared with the low fusimotor drive condition (A). Low frequency tremor did not appear to be consistently influenced, but if anything, was actually reduced in amplitude as fusimotor drive was increased. It should be noted that the amplitudes measured from our simulation should not be expected to precisely match those recorded experimentally. Of greater importance is the relative relationship between high and low frequency tremor amplitudes, and how they vary across individuals or simulation parameters. That said, our simulated amplitudes appear to be smaller than those recorded experimentally by a factor of about 10, which is reasonable considering the highly reduced/simplified nature of the model and the absence of any noise.

**Figure 7:**
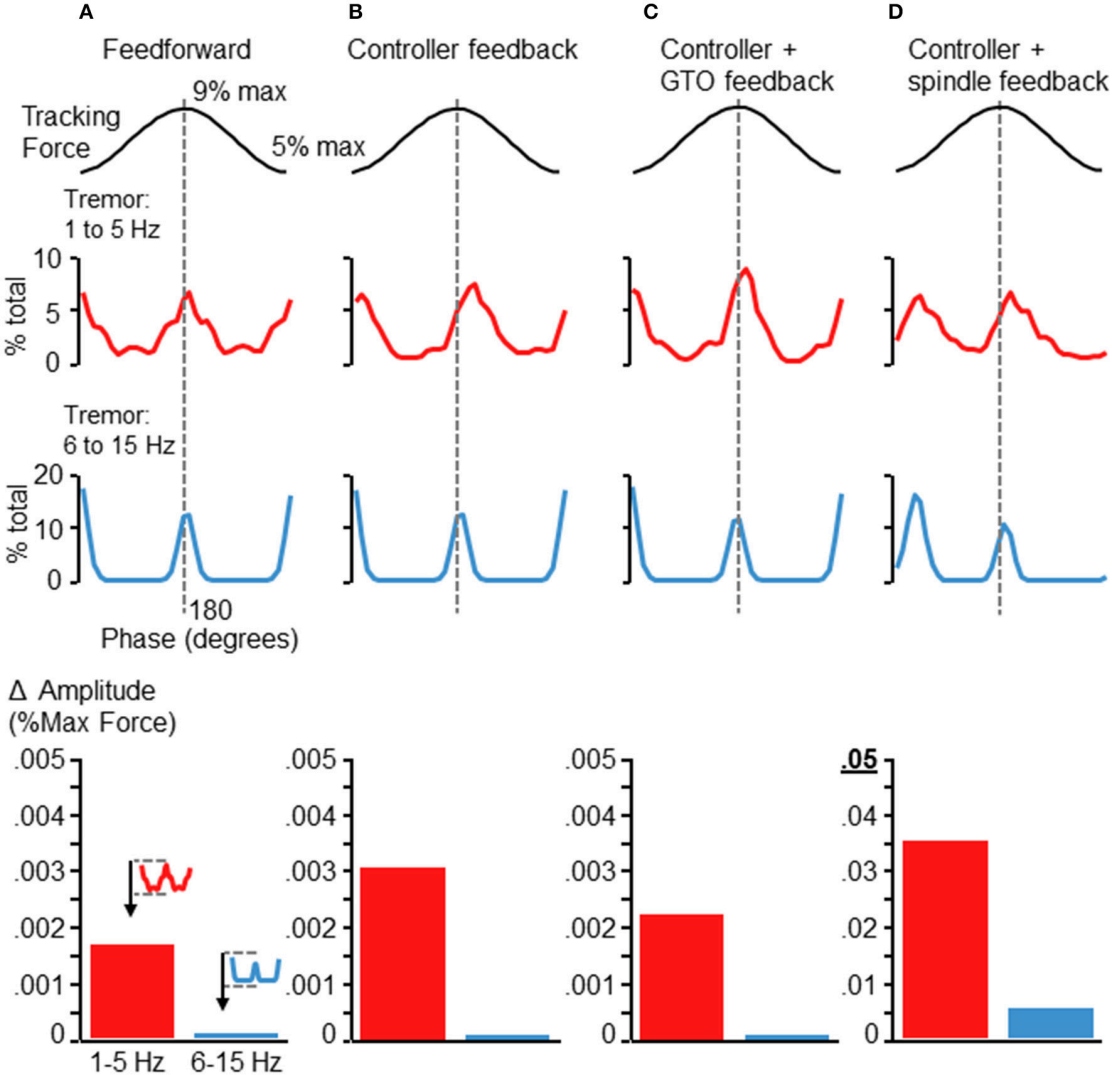
**Simulation results after removing sources of feedback control**. Each panels **(A–D)** depicts the results of the simulation after removing elements of the control loop. **(A)** Depicts the tremor modulation profile that emerged when the simulation was run completely feedforward, with no feedback. **(B)** Depicts the results when using the controller output, but no neural feedback. Panel **(C)** shows the tremor modulation occurring when Gogli tendon organ feedback was added to the controller, while panel **(D)** shows the tremor profile that emerged when only controller and spindle feedback were used. The baseline condition (before removal of feedback) was the same as in Figure 6A. Removal of the GTO feedback had minimal influence on the overall shape and timing of force fluctuations (compare Figure 6A with panel **D**). In contrast, elimination of spindle feedback not only reduced the magnitude of force tremor, but also influenced the general shape and timing of tremor modulation (panels **A–C**), particularly at the initiation of the rising phase of the target sinusoid. Shown at the bottom of each column are bar graphs depicting the average tremor amplitude modulation for each frequency band (as in Figures 5, 6). The addition of spindle feedback **(D)** to the model was the only condition which greatly modified tremor amplitudes. Note the change of scale for the bar graph in **(D)** as compared with **(A–C)**. While low frequency tremor modulation was increased by roughly a factor of 10 with the addition of spindle feedback, the high frequency tremor increased by about a factor of 50, with respect to any other condition.

We also ran the simulation after eliminating various elements of the control loop. Figure 7 shows the resulting tremor modulation pattern when the simulation was run completely feedforward (Figure 7A), using only feedback from the controller (Figure 7B), using only the controller and GTO feedback (Figure 7C), and using only the controller and spindle feedback (Figure 7D). Where spindle feedback is present, the fusimotor drive was set to 75 (as in Figure 6A). An increase in 6–15 Hz fluctuations occurred roughly at the peak and valley of the target sinusoid regardless of the feedback utilized in the control loop. However, the precise shape, timing, and magnitude of these fluctuations were altered by the type of feedback utilized. Inclusion of spindle feedback (Figure 7D) was necessary to produce realistic tremor variance patterns (compared with Figures 2, 5) at the initiation of the rising phase of the target sinusoid. Also it is worth noting that tremor amplitudes (bar graphs at bottom of Figure 7) were drastically reduced in the absence of spindle feedback roughly by a factor of 10 for 1–5 Hz tremor and by a factor of about 50 for 6–15 Hz tremor. These observations are well aligned with previous findings where reduction of afferent feedback was associated with reduced/eliminated physiological tremor (Halliday and Redfearn, 1958; Sanes, 1985; Erimaki and Christakos, 2008). Tremor modulation was in general particularly sensitive to spindle feedback, since increasing its effective gain through fusimotor drive (Figures 6B,C) or removing spindle feedback entirely (Figures 7A–C) both influence the overall shape, timing, and amplitude of tremor fluctuations. This occurred even though the largest change in muscle fascicle length was only about 1.1 mm. Interestingly, removal of GTO feedback had minimal effect (Figure 7D compared with Figure 6A).

## Discussion

In this study, we show that involuntary tremor can arise simply from the dynamic viscoelastic response of afferented muscles during voluntary production of isometric force. We characterized this tremor in two frequency bands as healthy adults performed a sinusoidal force tracking task. Furthermore, we simulated the spontaneous emergence of tremor from purely peripheral mechanisms using a computational closed-loop model comprised of well accepted musculotendon, spindle, and GTO computational modules. Our results extend the current understanding of how force variability arises independently of central mechanisms during production of isometric force. Importantly, our results suggest that simple force tracking tasks may provide a clinically and scientifically relevant window into the neural and mechanical factors which generate involuntary tremor.

Although tremor is not often attributed to the specific dynamics of voluntary force production, several investigations have suggested the existence, and potential importance, of such an interaction. For example, muscle stretch has been suggested to play a role in tremor modulation during dynamic force production. Specifically, declining isometric force is associated with at least some small degree of muscle fiber lengthening, toward resting state (Ito et al., 1998). Compared with shortening contractions, lengthening contractions are associated with increased force variability (Christou and Carlton, 2002) and increased motor unit coherence within the physiological tremor range (Semmler et al., 2002). However, it does not appear that muscle fiber lengthening, or associated spindle activity, could explain our results, since we would have expected a systematic and consistent increase in tremor during the descending phases of our sinusoidal target trajectory.

Similarly, in the production of bite force, 7–10 Hz jaw tremor is reduced in slowly increasing force ramps compared with constant or slowly decreasing force (Sowman et al., 2008). In the present study, a simple relation between force direction (increasing or decreasing) and finger tremor was not observed, likely due to differences in the physiology of bite vs. grip force control. Jaw tremor in the 7–10 Hz range depends upon the activity of periodontal mechanoreceptors (Sowman et al., 2006), and different bite-force dynamics may have led to different levels of dental intrusion (Schoo et al., 1983), and presumably adaptation of the periodontal mechanoreceptors (Sowman et al., 2008). Accordingly, both the dynamics of bite-force production and afferent feedback are important considerations when comparing healthy adults to those who suffer from bruxism (Laine et al., 2015b). While jaw tremor may depend on specific mechanical properties of the gums and their interaction with periodontal mechanoreceptors, these studies do demonstrate that tremor modulation can stem from afferent responses to dynamic force.

Stretch-reflex amplitudes have, in fact, been reported to change during the production of sinusoidal forces. For example, rhythmic (sinusoidal) pen-squeezing has been shown to produce stretch-reflex modulation in the FDS muscle (Xia et al., 2005). This is important in the context of the present study because oscillations of excitation around the stretch reflex loop are considered to be one of the major contributors to physiological tremor (Lippold, 1970; Young and Hagbarth, 1980; Christakos et al., 2006; Erimaki and Christakos, 2008). In the study of Xia et al. (2005), it was observed that stretch-reflex amplitudes were roughly modulated in a sinusoidal fashion such that increased reflex amplitudes were associated with higher background FDS EMG levels. A similar conclusion was reached by Stanislaus and Burne (2009), who reported a consistent relationship between stretch-reflex gain and overall contraction level regardless of force dynamics. If a similar sinusoidal modulation of reflex gain were responsible for tremor modulation in our study, we should have observed a sinusoidal modulation of tremor amplitude, which was not the case.

Few tasks involve only one muscle, as thus, it is possible that some tremors stem from an interaction among co-activated muscles. Due to the simplicity of our task and the posture of the hand, it is likely that any co-activated muscles were also co-modulated synergistically during tracking. It has been shown that synergistic muscles may share neural drive over a wide range of frequencies (Laine et al., 2015a). Moreover, we have shown that changes in the magnitude of an isometric fingertip force are likely produced by a simple scaling of a same muscle coordination pattern (Valero-Cuevas, 2000), while others have shown that isometric force magnitude does not influence the frequency content of shared neural drive among muscles of the hand (Poston et al., 2010). In addition to the potential for shared descending drive, neighboring co-activated muscles would likely show temporally-coordinated afferent activity and reflex responses as well. At the very least, it seems that the effects of slow sinusoidal contractions on musculotendon dynamics would be similar across all co-activated muscles, leading them to tremor at the same time relative to the slow voluntary action.

During voluntary force production, tremor may also stem from the recruitment, de-recruitment, and firing rates motor units. For example, the force level at which motor units are recruited can be lower than the force level at which they are de-recruited (De Luca et al., 1982), and due to the activation of persistent inward currents, the magnitude of neural drive needed to recruit a motor unit is often higher than the drive at which the same unit is de-recruited (Gorassini et al., 2002). Therefore, the population of motor units which generate a given force is partly determined by the recent contraction history of the muscle. Motor unit activity, especially the twitches of motor units near threshold, may contribute to isometric force tremor (McAuley and Marsden, 2000). The relevance of such mechanisms to the present study is not clear, but we can speculate that the contribution would be minimal, given the sufficiency of our simulation (which does not include firing motor units, their intrinsic properties, or any source of signal-dependent noise) to replicate the experimentally-observed tremor modulation. We would, however, assume that simulated tremor amplitudes would more closely match those observed experimentally if signal-dependent noise and/or intrinsic motor unit properties were included in our closed-loop system. This is a topic which certainly merits future investigation.

It is of course possible, even likely, that many of the above mentioned sources of force variability were still present to some degree in our study, but were not very consistent across participants or across target cycles. In that sense, they may explain some of the variation in tremor profiles observed across participants. Similarly, the degree to which any differences in muscle/tendon strength, size, and compliance across individuals would have influenced our results remains a topic for future investigation. We can speculate, however, that tendon strain magnitudes would likely be important, given that the dynamics of muscle stretch influence spindle output, which had the largest influence on tremor in the present study. The fact that our model could at least partly replicate cross-subject variation in tremor modulation through manipulation of fusimotor gains, which would be expected to vary across individuals (Ribot et al., 1986; Ribot-Ciscar et al., 2000, 2009; Hospod et al., 2007), adds validity to our simulation results and helps to mitigate concerns about its simplifications/assumptions. While it is beyond the scope of this study to precisely match the tremor profiles of every individual, or to exhaustively test the influence of all possible parameters, our results should serve as an important proof of principle upon which to base future investigation.

Despite the many possible sources of tremor within our task, it is clear that the dominant phase-dependent source of tremor was peripheral neuromechanical coupling, rooted in the viscoelastic properties of muscle and tendon. While smooth tracking is initially disrupted as a mechanical consequence of musculotendon dynamics, the spindle reflex system plays an important role in determining the overall magnitude and timing of the resulting tremor.

Our results, therefore, motivate and justify the development of similar experimental paradigms for scientific and clinical applications. For example, we propose that the tremor induced by slow voluntary force modulation may provide a simple measure of reflex integrity, providing an alternative to direct, yet time-consuming and often uncomfortable, perturbations of nerves or tendons. Further, it may be that characteristic patterns of tremor modulation would emerge within the context of spasticity, dystonia, or within conditions such as Parkinson's disease or essential tremor. As a means of probing peripheral neuromechanical coupling, the type of tremor described in this study may hold potential as a tool for understanding and assessing dysfunctional sensorimotor control in those with congenital or developmental disorders, or in those with acquired dysfunction due to trauma or disease. Finally, neuromechanical coupling may contribute mechanistically to the maintenance or amplification of pathological tremor. While we did not test the effects of inserting a descending tremor-frequency input into our simulation, such investigation may be informative, and perhaps even suggest novel avenues for clinical intervention.

## Author contributions

CL, AN, and FV contributed to the design, execution and drafting of this work, and approved the final manuscript. Experimental data was collected by CL and simulations were implemented by AN.

## Funding

Research reported in this publication was supported by the National Institute of Arthritis and Musculoskeletal and Skin Diseases of the National Institutes of Health under Awards Number R01 AR-050520 and R01 AR-052345. The content is solely the responsibility of the authors and does not necessarily represent the official views of the National Institutes of Health.

### Conflict of interest statement

The authors declare that the research was conducted in the absence of any commercial or financial relationships that could be construed as a potential conflict of interest.
